# MG-Digger: An Automated Pipeline to Search for Giant Virus-Related Sequences in Metagenomes

**DOI:** 10.3389/fmicb.2016.00428

**Published:** 2016-03-31

**Authors:** Jonathan Verneau, Anthony Levasseur, Didier Raoult, Bernard La Scola, Philippe Colson

**Affiliations:** ^1^Aix-Marseille University, URMITE UM 63 CNRS 7278 IRD 198 INSERM U1095Marseille, France; ^2^IHU Méditerranée Infection, Assistance Publique – Hôpitaux de Marseille, Centre Hospitalo-Universitaire Timone, Pôle des Maladies Infectieuses et Tropicales Clinique et Biologique, Fédération de Bactériologie-Hygiène-VirologieMarseille, France

**Keywords:** metagenomes, giant virus, *Megavirales*, bioinformatics, pipeline, mimivirus

## Abstract

The number of metagenomic studies conducted each year is growing dramatically. Storage and analysis of such big data is difficult and time-consuming. Interestingly, analysis shows that environmental and human metagenomes include a significant amount of non-annotated sequences, representing a ‘dark matter.’ We established a bioinformatics pipeline that automatically detects metagenome reads matching query sequences from a given set and applied this tool to the detection of sequences matching large and giant DNA viral members of the proposed order *Megavirales* or virophages. A total of 1,045 environmental and human metagenomes (≈ 1 Terabase) were collected, processed, and stored on our bioinformatics server. In addition, nucleotide and protein sequences from 93 *Megavirales* representatives, including 19 giant viruses of amoeba, and 5 virophages, were collected. The pipeline was generated by scripts written in Python language and entitled MG-Digger. Metagenomes previously found to contain megavirus-like sequences were tested as controls. MG-Digger was able to annotate 100s of metagenome sequences as best matching those of giant viruses. These sequences were most often found to be similar to phycodnavirus or mimivirus sequences, but included reads related to recently available pandoraviruses, *Pithovirus sibericum*, and faustoviruses. Compared to other tools, MG-Digger combined stand-alone use on Linux or Windows operating systems through a user-friendly interface, implementation of ready-to-use customized metagenome databases and query sequence databases, adjustable parameters for BLAST searches, and creation of output files containing selected reads with best match identification. Compared to Metavir 2, a reference tool in viral metagenome analysis, MG-Digger detected 8% more true positive *Megavirales*-related reads in a control metagenome. The present work shows that massive, automated and recurrent analyses of metagenomes are effective in improving knowledge about the presence and prevalence of giant viruses in the environment and the human body.

## Introduction

The first giant virus of amoeba, Mimivirus, was isolated in 2003 from a water sample by co-culturing on *Acanthamoeba polyphaga*, a strategy implemented to find *Legionella*-like bacteria (La Scola et al., 2003; Raoult et al., 2007). The Mimivirus DNA genome was found to harbor ≈1.2 megabase pairs (Mbp) and 1,000 genes, including some which had never previously been detected in viruses, such as those encoding for translation apparatus proteins (La Scola et al., 2003; Raoult et al., 2004). Subsequently, over the past decade, dozens of new giant viruses have been discovered, essentially from environmental samples (mostly water and soil; Colson and Raoult, 2012; Pagnier et al., 2013; Legendre et al., 2014). They comprised new viral families, including *Mimiviridae* (La Scola et al., 2008; Pagnier et al., 2013) and *Marseilleviridae* (Boyer et al., 2009; Colson et al., 2012b; Pagnier et al., 2013), and two new putative viral families including pandoravirus isolates (currently the largest known viruses; Philippe et al., 2013), and *Pithovirus sibericum* (Legendre et al., 2014). These giant viruses were related to the group of nucleocytoplasmic large DNA viruses (NCLDVs) described since 2001 as being composed of five viral families: *Ascoviridae, Asfarviridae, Iridoviridae, Phycodnaviridae*, and *Poxviridae*, whose members infect a wide variety of eukaryotic hosts (Iyer et al., 2001; Yutin et al., 2009). Reclassifying all these giant viruses into a new viral order (*Megavirales*) has recently been proposed (Colson et al., 2013a). Indeed, their common origin has been inferred from the results of phylogenetic and phyletic analyses, and they share common virion architecture and major biological features, including reproduction within cytoplasmic factories. Giant viruses of amoeba are the largest megaviruses. The size of these virions and their gene complements has changed our view of the viral world and its diversity, and has called into question the definition and classification of viruses (Raoult and Forterre, 2008; Colson et al., 2012a; Raoult, 2014).

*Megavirales* members, including amoebal giant viruses, have been shown over the past decade to be very common in our biosphere (Colson and Raoult, 2012; Pagnier et al., 2013). Concurrently, Mimivirus has been increasingly associated with pneumonia. Thus, various serological studies have shown higher rates of exposure to this virus among people with pneumonia compared to controls (La Scola et al., 2005; Raoult et al., 2007; Bousbia et al., 2013). An experimental model showed that Mimivirus can cause pneumonia in mice (Khan et al., 2007) and, recently, two mimiviruses were isolated from patients with unexplained pneumonia (Colson et al., 2013c; Saadi et al., 2013a,b). In addition, marseilleviruses were isolated from the stool of a young Senegalese man and from a blood donor, and were detected by fluorescence *in situ* hybridization (FISH) in the lymph node of a young child with lymphadenitis and by PCR from his serum, as well as from blood donors and multi-transfused patients (Lagier et al., 2012; Colson et al., 2013b; Popgeorgiev et al., 2013a,b). Antibodies to marseilleviruses were also detected in various populations, including in healthy adults (Colson et al., 2013c; Mueller et al., 2013). Thus, overall, giant amoebal viruses are common in our environment and their presence is detected in humans, which raises the question of their pathogenicity.

Concurrently, giant virus-related sequences have been detected in environmental and human metagenomes (Monier et al., 2008; Loh et al., 2009; Kristensen et al., 2010; Colson et al., 2013b; Law et al., 2013). Moreover, the size of giant viruses probably contributed to their neglect because most of the samples studied for the presence of viral sequences were filtered prior to their analysis in order to remove bacteria and eukaryotes and to work on the ultrafiltrate (Colson et al., 2013b). Metagenomics is a new method that developed over the past 12 years alongside the discovery of giant amoebal viruses, and was largely boosted by the advent of high-throughput sequencing technologies. This approach relies on massive sequencing of all the DNA present in a given sample without any culture isolation of the microorganisms performed beforehand (Handelsman, 2004; Mokili et al., 2012). With next-generation sequencing (NGS) technologies, the time and cost of genome sequencing has fallen dramatically. Big data generated by these new technologies, and thus needing to be stored and analyzed, is considerable, reaching over one Terabp (Tbp) per run (Shendure and Ji, 2008). Moreover, the number of metagenomic studies conducted each year is growing. Storage and analysis of these data can be difficult due to their size and the time required for analyses, even for powerful computers and softwares. Lots of softwares have been described for analysing metagenomic data including, for instance, Metavir (Roux et al., 2014), metAMOS (Treangen et al., 2013), MG-RAST (Glass et al., 2010), and VIROME^1^. However, these tools, although usually sophisticated, have been found to display one or more among the following limitations: availability, installation on our bioserver for stand-alone use on both Linux and Windows operating systems, ease of use, analysis of customized query and target sequence databases, setting of parameters for sequence similarity searches, automation, information provided in output files, and ability to handle large sequences sets.

Because there is growing interest in giant viruses in the field of evolutionary biology and in environmental and clinical virology, it is necessary to systematically and repeatedly search in metagenomes for sequences from these viruses or close relatives. For this purpose, we established a database of ready-to-use metagenomes and a bioinformatics pipeline which detects reads in these metagenomes that are the most similar to sequences from a given set. We used this tool, which we entitled ‘MG-Digger’ because it allows to dig into metagenomes to identify reads of interest, to detect metagenome sequences matching those from *Megavirales* representatives.

## Materials and Methods

### Database of Metagenomes

#### Type of Metagenomes

The database of metagenomes was created with both environmental and human metagenomes. Control metagenomes, required to validate the functionality and effectiveness of our tool, consisted of metagenomes previously described as containing giant virus-related sequence reads. They comprised metagenomes from sewage and human sera (Loh et al., 2009), from the plasma of patients with liver diseases (Law et al., 2013) and from water samples from the Indian Ocean (Williamson et al., 2012). In addition, 16 soil metagenomes recently investigated for the presence of giant virus sequences (Kerepesi and Grolmusz, 2015b) were analyzed.

#### Source of the Collected Metagenomes

The metagenomes were downloaded from multiple sources, including the National Center for Biotechnology Information (NCBI)^2^, the metagenome platform MG-RAST^3^, and the Genomes OnLine database (GOLD)^4^.

#### Type of Metagenome Files

Metagenome sequence files were in various formats, including FASTA, FASTQ, and SRA (for Sequence Read Archive). It was therefore necessary to unify all these formats into a single one so that the sequences of reads (sequencing product of a size ranging between ≈60–400 nucleotides) were themselves in the same format. The SRA of the NCBI stores metagenomes from scientific projects after their compression and incorporates an accession number. The advantage of this format is data compression, up to a factor of five, while conserving sequencing data. SRA files can be converted into FASTA and FASTQ files using the bioinformatics tools developed by NCBI in the SRA tools’ package^5^.

### Genomes of Giant Viruses

The sequences used as queries to search for related sequences in metagenomes were nucleotide (genomes and genes) and protein (putative gene products) sequences from the members of the proposed order *Megavirales*, including asfarviruses, ascoviruses, iridoviruses, poxviruses, phycodnaviruses, mimiviruses, marseilleviruses, pandoraviruses, *P. sibericum*, faustoviruses, and from the mimivirus virophages. All these sequences are available from the NCBI GenBank sequence databases with the exception of the sequences of five faustoviruses other than Faustovirus strain E12, which were isolated in our laboratory and whose genomes were not available from GenBank at the time of the analysis (Reteno et al., 2015). The risk of incorrectly annotating a metagenomic read based on wrong information deposited in the query sequence database was very limited, because most of the sequences of megaviruses and virophages had been analyzed often over the past decade by several teams and sequence sets from giant viruses of amoeba and virophages were manually curated.

### MG-Digger Implementation and Configuration

We used the Python language, which is widely used in bioinformatics for programming. The advantages of this language include open access, potential multi-platform use (Windows, Unix, MacOS), simple syntax that makes it highly accessible compared to other programming languages, and very complete libraries. A library is a set of functions, which are gathered and made available. The Biopython library, for instance, manipulates biological and bioinformatics data, and is considered a script language.

BLAST+ (Camacho et al., 2009) is a similarity search tool for nucleotide or protein sequences developed by the NCBI. BLAST+ runs the Basic Local Alignment Search Tool (BLAST; Altschul et al., 1990) in a local server with an existing database. This database can be created with the makeblastdb application available in BLAST+, and in this case was built from our megavirus and virophage sequence database. BLAST+ makes similarity searches possible without internet access and can be used by employing command lines. It can be easily integrated into a pipeline. BLAST+ has the significant advantage of being able to be used on a multiprocessor server, which decreases the time taken for analyses while increasing the number of processors used. Nevertheless, the nr (protein) and nt (nucleotide) sequence databases used by this tool are very large (≈90 Gbp), which requires assigning ≈105 GB for them. In addition, these databases must be periodically updated to analyze the latest sequences published.

### MG-Digger Availability

MG-Digger is freely available for Linux and Windows at the following URL^6^.

### Statistical Analyses

Proportions were compared with a corrected chi-square test using the OpenEpi epidemiologic calculator v.3.03a^7^; *P*-values < 0.05 were considered to be statistically significant. Agreement between tools was performed using the Cohen’s kappa test.

## Results

### Giant Virus Database

The nucleotide (genes and genomes) and protein (putative gene products) sequences were obtained for a total of 93 *Megavirales* members and five virophages (Supplementary Table S2). The *Megavirales* members included amoebal giant viruses with seven mimiviruses, three marseilleviruses, two pandoraviruses, *P. sibericum*, and six faustoviruses. This giant viral sequences database can be completed and updated at any time to include newly described genomes or partial sequences.

### Bioinformatics Pipeline Operation

**FIGURE 1:**
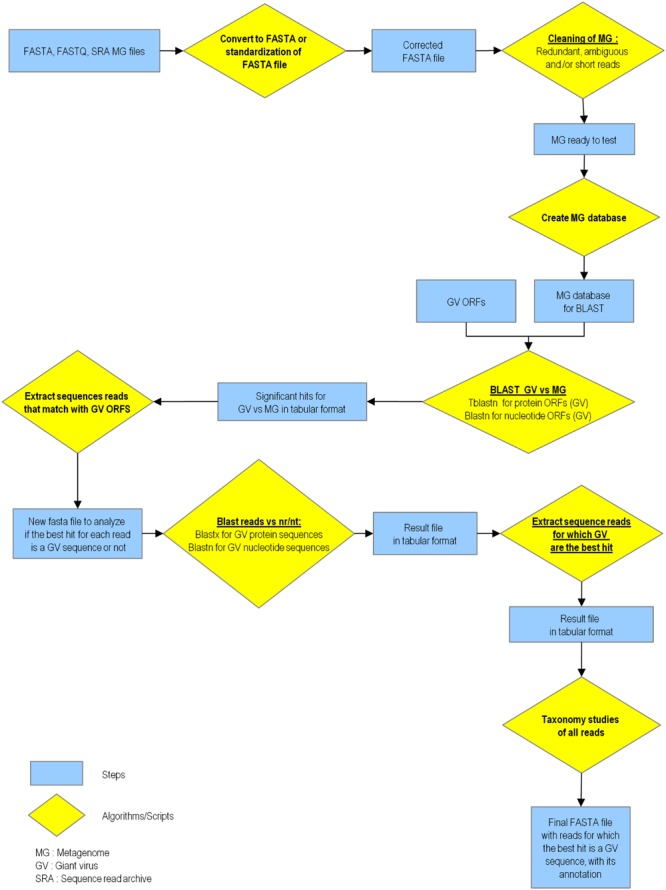
**Flowchart of the MG-Digger tool**.

The pipeline dedicated to the search for giant virus-related sequences in metagenomes comprises several scripts written in Python language and include independent modules (**Figure 1**). These modules automatically operate successively, without the need for any user intervention. Alternatively, the user can launch a single module to perform only part of the analysis. The type of BLAST analysis performed by the pipeline can be chosen, depending on the nature of the sequence set to study. Hence, BLAST analyses that are launched can use nucleotide or protein queries and target sequences.

**FIGURE 2:**
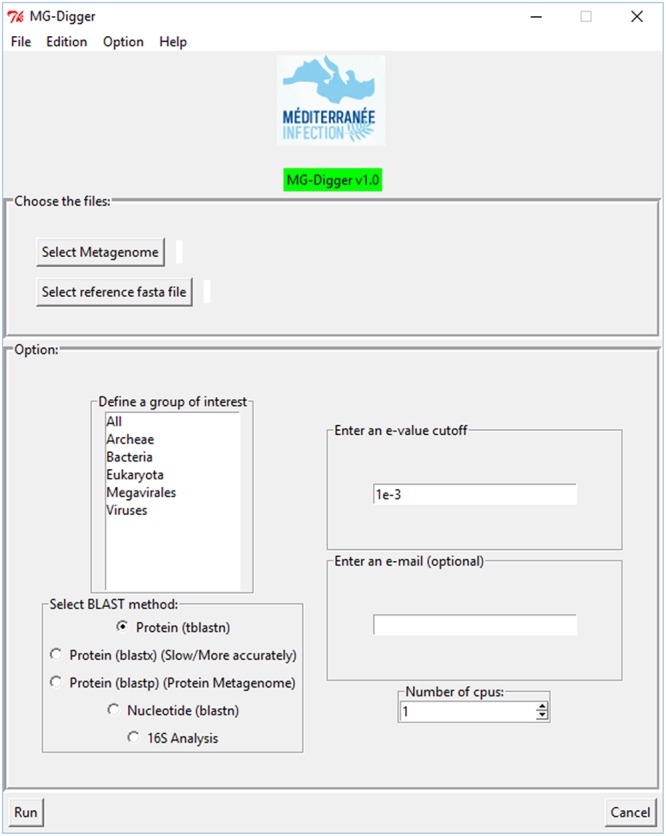
**Screenshot of MG-Digger graphical user interface**.

MG-Digger is freely downloadable for Linux and Windows^8^ as a compressed folder. This folder contains a tutorial (README.txt file) with instructions on installing and using MG-Digger. It also contains an executable file that opens a user-friendly graphic interface (**Figure 2**) and a configuration file that sets the path to the local BLAST database from this interface. This interface was implemented to speed up and make it easier for non-bioinformaticians to use MG-Digger. MG-Digger only requires the additional installation of Biopython on the user’s computer^9^, Python 2.7.x^10^ and BLAST+ version 2.2.28 or later^11^ to operate. It can operate on standard desk computers, as the minimum requirements are a 1 GHz 32- or 64-bit processor, 1 GB of RAM and 120 Go of hard disk space. For easier access for our research laboratory community and faster processing, MG-Digger has been installed on the URMITE bioserver.

In short, the operation of the pipeline includes three main steps (**Figure 1**). In the first step, metagenome FASTA, FASTAQ, or SRA files that have been downloaded from any source are converted into standard FASTA files. Each of these files are then ‘cleaned,’ which means that all redundant, short and ambiguous sequences are removed to improve the performance of analyses and reduce the time it takes. Default options for this cleaning are to eliminate reads that appear in multiple copies, reads that are shorter than 40 nucleotides, and reads that involve more than 20% of ambiguities (indeed, the nature of the nucleotides determined by NGS is uncertain, and replaced by an ‘N’ at least at 20% of the read positions). All these parameters are adjustable to obtain a metagenome with the appropriate basic criteria. In addition, the metagenome sequence set can be completed and updated at any time. In the second step, a metagenome database is created and metagenomic reads that show significant similarity with sequences from giant viruses are retrieved by a tBLASTn search when protein sequences are used as query, or a BLASTn search when genes or genomes are used as query, using BLAST+ (Camacho et al., 2009). In the third step, these reads are tested by BLAST against the GenBank nr or nt sequence database [plus any additional sequences; here, the sequences of five unpublished faustoviruses obtained in our laboratory were added (see Supplementary Table S2)]. Such reciprocal BLAST hit strategies are widely used for the identification of orthologs (Jordan et al., 2002; Li et al., 2003). Reads are considered as related to giant viruses or virophages if their best ‘hit’ is a giant virus or virophage sequence (default significance threshold being an *e*-value of 10^-5^). The output of this pipeline consists of four simple files: first, the raw BLAST output tab-delimited file for reads matching a query sequence; second, a BLAST output file for metagenomic reads for which the best hit is possibly a query sequence; third, the FASTA file of reads for which the best BLAST hit is possibly a query sequence; and fourth, a file with analyses data that includes metagenome identification, numbers of metagenomic reads processed, extracted and found to have as best hit one or several query sequences, the identification of these query sequences, the duration of analyses and number of central processing units (CPU) used. Selected reads can be stored and eventually used to conduct assemblies, multiple alignments or phylogenetic trees.

### Metagenome Database

A total of 1,045 metagenomes were collected and stored on our server in FASTA format after having been processed with MG-Digger. They were generated from 227 environmental samples from 21 studies and 818 human samples from 17 studies (Supplementary Table S1). The total size of the metagenome database corresponded to approximately 1 Tbp, and was comprised of files whose total sequence lengths ranged from 5 Mbp to 12 Gbp per study. These files were prepared for further analysis by MG-Digger and their size had been reduced by MG-Digger. For instance, for control metagenomes, the amount of sequences was reduced by 53%, decreasing from 20.2 to 9.6 Gbp.

### Validation of the Pipeline Performance on Control Metagenomes

**FIGURE 3:**
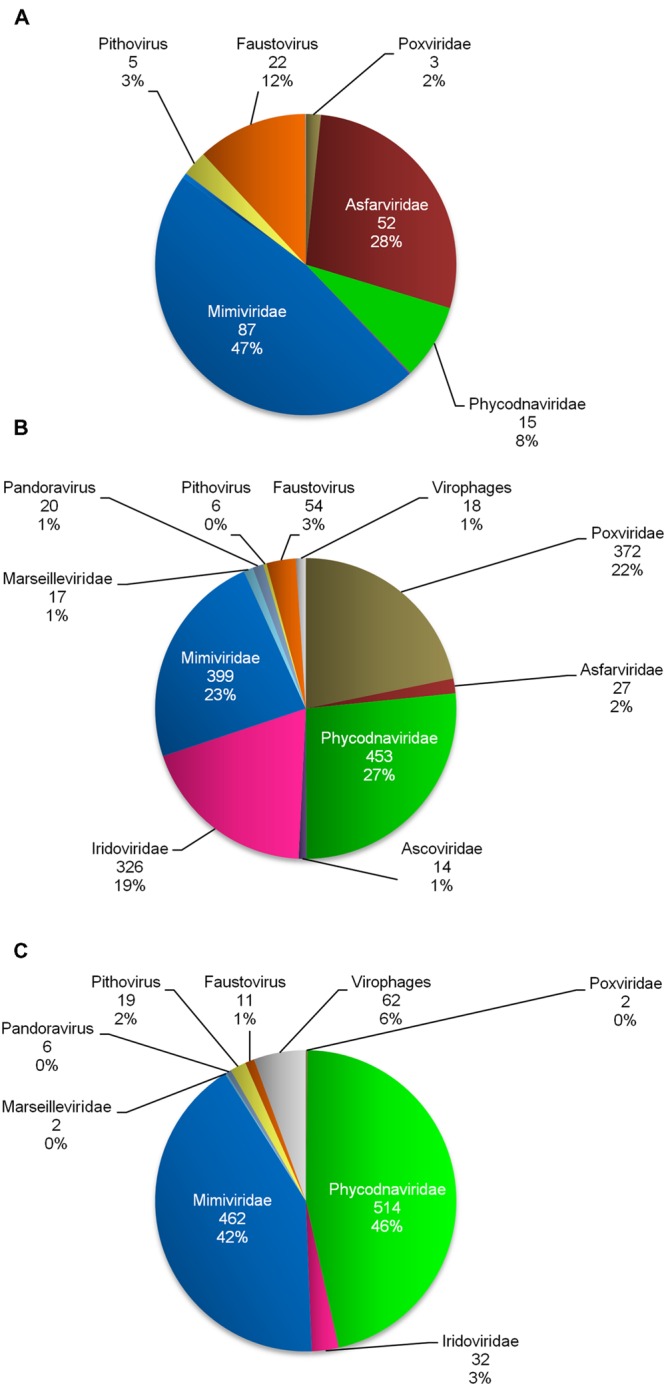
**Distribution of *Megavirales* member- and virophage-related reads identified by MG-Digger in metagenomes generated from human sera and sewage (**A**; Loh et al., 2009), plasma from patients with liver diseases (**B**; Law et al., 2013) and water from the Indian Ocean (**C**; Williamson et al., 2012)**.

To assess the functionality of the pipeline, it was tested on published metagenomes, which were found to contain megavirus-like sequences. These analyses included sequences from 93 *Megavirales* representatives and five virophages as a query. In the metagenome described by Loh et al. (2009) obtained from human serum samples and sewage, the number of reads showing a significant similarity with a giant virus sequence was 185 out of 29,117 (0.6%; **Figure 3A**). These reads were found to have as the best match a mimivirus in 87 cases, an asfarvirus in 52 cases, a faustovirus in 22 cases, a phycodnavirus in 15 cases, *P. sibericum* in five cases, and a poxvirus in three cases. These results substantially expand the number of megavirus-related reads obtained by Loh et al. (2009), who only searched for asfarvirus-like sequence reads using BLASTx. In the metagenome described by Law et al. (2013) obtained from plasma samples from humans with liver disease, the number of reads showing a significant similarity with giant virus sequences was 1,706 out of 42,706,883 (0.004%; **Figure 3B**); in this study, BLASTx search against the GenBank nr database was performed with an *e*-value cutoff of 1*e* – 5 after assembly of reads. The best hit for these 1,706 reads was a phycodnavirus in 453 cases, a mimivirus in 399 cases, a poxvirus in 372 cases, an iridovirus in 326 cases, a faustovirus in 54 cases, an asfarvirus in 27 cases, a pandoravirus in 20 cases, a marseillevirus in 17 cases, an ascovirus in 14 cases, *P. sibericum* in six cases, and virophages in 18 cases. The predominance of phycodnavirus-related reads found here is consistent with findings by Law et al. obtained through BLAST analysis. In contrast, however, our analysis identified a greater number of iridovirus-like sequences. It is noteworthy that in these two analyses, sequences related to amoebal giant viruses whose sequences were not available at the time of publication of the initial analyses, including pandoraviruses, *P. sibericum* and faustoviruses, were detected.

**FIGURE 4:**
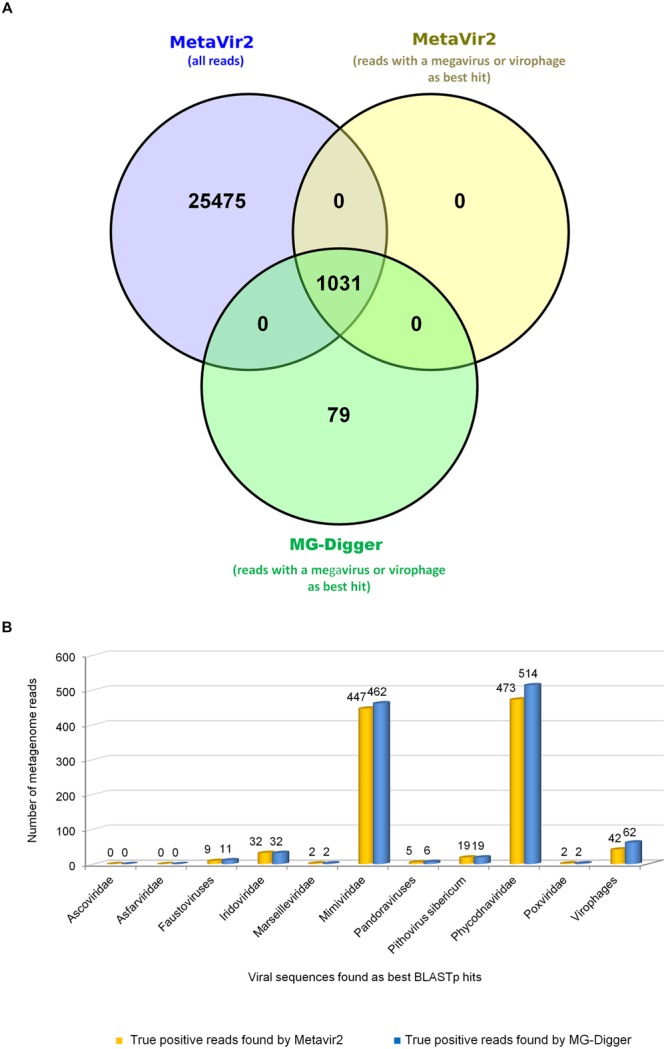
**Comparison of the MG-Digger and METAVIR 2 results for the environmental metagenome dataset generated in Williamson et al.’s study (Williamson et al., 2012). (A)** Venn diagram of number of reads identified as having a *Megavirales* member or virophage sequence as best hit by the two tools. **(B)** Distribution of hits according to the viral families found as best hit by the two tools.

We also performed our analysis on the metagenomes of Williamson et al. (2012), which had already been processed using the well-known Metavir 2 tool (Roux et al., 2014). Using MG-Digger, the number of reads showing significant similarity (*e*-value threshold, 1*e* – 3) to *Megavirales* members and virophages was 1,110 (1,048 and 62, respectively), out of 1,636,697 (0.07% overall; **Figure 3C**). Among them, we found reads having as the best hit a phycodnavirus in 514 cases, a mimivirus in 462 cases, an iridovirus in 32 cases, *P. sibericum* in 19 cases, a faustovirus in 11 cases, a pandoravirus in six cases, a marseillevirus in two cases and a poxvirus in two cases. These results are somewhat similar to those reported by Williamson et al. (2012) using APIS (Automated Phylogenetic Inference System) and BLASTp as tools, which showed a predominance of phycodnavirus-related reads followed by mimivirus-related reads. Regarding the analysis we conducted using Metavir 2, we downloaded the file from the Metavir website^12^ that contains the reads identified by this software using BLASTx comparison with the NCBI Refseq complete viral genomes protein sequences database (with 1*e* – 3 and 50 as *e*-value and bit-score thresholds, respectively). We then selected reads best matching megaviruses or virophages by checking the identification of these best matches in GenBank using their gi, and found 26,506 reads (**Figure 4A**). Finally, we performed a BLASTx search (*e*-value threshold, 1*e* – 3) against the NCBI GenBank non-redundant protein sequence database (nr; same version available in November 2015 as that used with MG-Digger) to check whether best hits were megaviruses or virophages, and we found only 1,031 reads that fulfilled this criterion. Of these reads, 473, 447, 32, 19, 9, 5, 2, and 2 had as best hits phycodnaviruses, mimiviruses, iridoviruses, *Pithovirus sibericum*, faustoviruses, pandoraviruses, marseilleviruses, and poxviruses, and 42 reads had as best hits virophages (**Figures 4A,B**). MG-Digger detected all the 1,031 reads detected by Metavir 2 as having a *Megavirales* member or virophage sequence as the best hit. In addition, MG-Digger identified 79 additional reads to the set recovered by Metavir 2, i.e., 8% more; best matches for these reads were related to phycodnaviruses (*n* = 41), mimiviruses (15), virophages (20), faustoviruses (2), and pandoraviruses (1). This difference may be due to different strategies for selecting the reads that match giant viruses and virophages (BLASTx for Metavir 2 and tBLASTn for MG-Digger) or to differences in viral set used (the RefSeq viral protein sequences database for Metavir 2 and a customized database of megaviruses and virophages for MG-Digger). Overall, the proportion of metagenomic reads identified as having a megavirus or a virophage as best BLAST hit tended to be higher using MG-Digger than Metavir 2 (*p* = 0.09). Nevertheless, both tools showed an excellent agreement (*k* = 0.963; Cohens’ kappa test), and no significant difference was noted for any of the viral or putative families. In contrast, using raw data provided by Metavir would have led to a 24-fold overestimation of the proportion of these viruses in the metagenomes. These results suggested that MG-Digger may be slightly more sensitive and is more specific than Metavir in terms of identifying *Megavirales* or virophage-related reads in metagenomic datasets. In addition, for 14, seven and three reads found using MG-Digger in the metagenomes described by Williamson et al. (2012; with an *e*-value threshold of 1*e* – 3), sequences from the recently described Yellowstone Lake virophages, *P. sibericum*, and Faustovirus were the only significant hits. In terms of reads whose best match was *P. sibericum*, the mean ± standard deviation (SD) for amino acid identity and alignment length were 38 ± 5% and 105 ± 36 amino acids, respectively, and mean ± SD coverage of sequence reads by the amino acid alignments was 71 ± 22% (range, 37–98). Notably, two reads had as the only match an ATP-dependent DNA ligase (YP_009001365.1) and a Ser/Thr protein kinase (YP_009001306.1) from *P. sibericum*, while three reads matched a topoisomerase IIA (YP_009001040.1) from this virus (Supplementary Figures S1 and S2).

Finally, we searched for sequences matching those from giant viruses and virophages in 16 soil metagenomes recently investigated by Kerepesi and Grolmusz (2015b), using a tool described in 2015. We detected a total of 1,150 reads among 11,2674,624 whose best match was a megavirus (*n* = 1,146) or a virophage (*n* = 4) sequence, which is 10.7-fold greater and a significantly higher proportion of the total number of metagenomic reads (*p* < 1*e* – 6) than with the ‘Giant Virus finder’ tool, which performs a BLASTn search as a first step whereas MG-Digger performs an initial tBLASTn search (Supplementary Figure S3). In 36 and 15 cases, the best hits were sequences from Faustovirus and *P. sibericum*, respectively.

The approximate duration of our analyses conducted on these control metagenomes using 40 of 192 CPU available in our bioserver (SGI Altix UV100 calculator) and 2 GB of RAM was 9 h, 2.5 days, and 4.5 days for the metagenome studied by Loh et al. (2009), Williamson et al. (2012), and Law et al. (2013), respectively, and it ranged between 4 and 11 h for the 16 metagenomes analyzed by Kerepesi and Grolmusz (2015b). The length of these analyses was not compared to that of other tools, as approximate duration was only given in the study by Kerepesi and Grolmusz (2015b) and such a comparison would have implied using computers with same characteristics and performance.

## Discussion

MG-Digger, a user-friendly computational tool implemented in our laboratory for the detection of *Megavirales*-like or virophage-like sequences in metagenomes, automatically generated ready-to-analyze metagenome files and annotated 100s of sequences as significantly matching those of giant viruses or virophages. These findings suggest the potential presence of megaviruses, virophages or close relatives in the original samples. MG-Digger functionality and efficiency to detect the best matches of a giant viral sequence set was validated by analyzing environmental or human metagenomes that were previously analyzed in four studies using other tools. Accurate comparisons of the results of MG-Digger with those previously obtained with other tools that analyze viral metagenomes are difficult because these tools used different strategies consisting of different similarity searches with different virus sequence databases. However, a comparison with results obtained for an Ocean Water metagenome by Metavir 2 (Roux et al., 2014), a widely used tool for the study of viral metagenomes, suggested that MG-Digger had similar or slightly greater sensitivity. Moreover, MG-Digger presents several advantages in comparison with previously described tools. Thus, MG-Digger performs a fully automated process that starts with downloaded raw metagenomes and ends by providing annotations for metagenome sequences, and it operates as a standalone software either on a personal computer or on a laboratory bioserver, which enables large sequence sets to be handled within a limited time. In addition, MG-Digger requires limited user computer skills, as a graphical user interface is implemented that operates by clicking a computer mouse button, using either Linux or Microsoft Windows operating systems. Moreover, it generates ready-to-analyze metagenomes for any subsequent searches, and can handle customized query and target sequence databases, using adjustable parameters for sequence similarity searches. Finally, output files contain selected reads with the identification of their best match. MG-Digger has been made available to any student or senior investigator in our clinical and research microbiology laboratory and is currently used for several studies of metagenomic datasets.

Metagenome sequences identified in the present work were most often similar to phycodnavirus or mimivirus sequences. However, matches were also obtained with sequences from giant members belonging to putative new viral families in the proposed order *Megavirales*, namely pandoraviruses, *Pithovirus sibericum* and faustoviruses. The results of the present work show that massive and automated analysis of metagenomes can identify some sequences as most closely related to newly described organisms, and some non-annotated sequences (which represent a ‘dark matter’) as only related to these newly described organisms. Such analyses, therefore, increase our level of knowledge about the presence and prevalence of these viruses, firstly in the environment, which supports possible human exposure, and secondly in humans. Pandoravirus-related sequences have been recently reported in metagenomes generated from various soil samples worldwide (Kerepesi and Grolmusz, 2015a), and a tool (named the Giant Virus finder) was described by the same team and applied to the search for giant viral sequences in environmental metagenomes, which illustrates the rising interest in these giant viruses in the field of environmental microbiology (Kerepesi and Grolmusz, 2015b). In addition, best matches with faustovirus sequences were found by our team in metagenomes generated from Mississippi ponds and from the serum samples of healthy Egyptian volunteers (Reteno et al., 2015). In contrast, to our best knowledge, *P. sibericum*-related sequences were detected here for the first time from an environmental or a human metagenome. This suggests that relatives of *P. sibericum*, which has been described as having been retrieved from Late Pleistocene Siberian permafrost (Legendre et al., 2014), currently exists and could be isolated in the near future from current samples. Hence, it is worth noting that the inclusion in our query dataset of sequences belonging to new putative viral families, such as pandoraviruses, *P. sibericum* or faustoviruses, detected matches with these viruses. This suggests that such searches should be updated frequently to take into account the expanding diversity of *Megavirales* and, concurrently, the increasing amount of metagenomic data. For this purpose, user-friendly automated pipelines such as MG-Digger should be used recurrently to study the prevalence of old and new *Megavirales* representatives in the environment and in humans, and to gain a better understanding of their presence and possible pathogenicity in humans. Metagenome sequences detected by such an approach can only be considered as the closest match to a previously identified sequence and do not necessarily belong to the same organism or a similar organism. Such searches could be complemented by molecular detection of the viral sequences which are most represented in the samples from which the metagenomes were generated, when they are available, or by culture isolation, as previously successfully performed for Senegalvirus, a marseillevirus (Colson et al., 2013b).

Analyses conducted by MG-Digger provided additional evidence that *Megavirales* relatives are common in our biosphere and in humans (Colson et al., 2013b; Pagnier et al., 2013). Particular emphasis should be given to amoebal giant viruses that were found here to be the best matches for metagenome sequences generated from human samples that were collected from healthy individuals and from patients with infectious or non-infectious hepatitis. The recent isolation of giant amoebal viruses in human samples is an emerging field in human virology (Colson et al., 2013c). Until now, only members from the *Mimiviridae* and *Marseilleviridae* families have been detected and isolated in human samples. LBA111 and Shan virus are mimiviruses that were isolated from bronchoalveolar fluid and stool samples, respectively, from two Tunisian pneumonia patients (Saadi et al., 2013a,b). Senegalvirus and Giant Blood Marseillevirus are two marseilleviruses that were serendipitously discovered after detection of Marseillevirus-like sequences in the stool of a healthy Senegalese man and blood from a blood donor in Marseille, respectively (Lagier et al., 2012; Popgeorgiev et al., 2013a). A marseillevirus was subsequently detected in a lymphadenitis (Popgeorgiev et al., 2013b). There is a strong body of evidence that mimiviruses might cause pneumonia, while the pathogenic role of marseilleviruses in humans remains to be deciphered (Colson et al., 2013c). In addition, *Acanthocystis turfacea* chlorella virus 1, a phycodnavirus, was recently found by metagenomics in human oropharyngeal samples and has been associated with cognitive disorders (Yolken et al., 2014). These data are an incentive to continue searching for sequences that match giant viruses in metagenomes generated from human samples in order to assess the link between megaviruses and humans.

Finally, although MG-Digger was basically designed for the detection of giant virus sequences, query sequence sets can be selected according to the objectives. Thus, our tool could be used to search for matches with sequences related to bacteria, eukaryotes, archaea and other viruses in metagenomes.

## Author Contributions

PC, AL, DR, and BLS designed the study. JV and PC performed the analyses. PC, AL, DR, BL, and PC analyzed and discussed the results. JV, AL, and PC wrote the manuscript.

## Conflict of Interest Statement

The authors declare that the research was conducted in the absence of any commercial or financial relationships that could be construed as a potential conflict of interest.

## Footnotes

1 http://virome.dbi.udel.edu/

2 http://www.ncbi.nlm.nih.gov/Traces/sra/

3 http://metagenomics.anl.gov/

4 http://www.genomesonline.org/

5 http://www.ncbi.nlm.nih.gov/Traces/sra/?view=software

6 http://www.mediterranee-infection.com/index.php

7 www.OpenEpi.com

8 http://www.mediterranee-infection.com/index.php

9 http://biopython.org/wiki/Download

10 https://www.python.org/downloads/

11 ftp://ftp.ncbi.nlm.nih.gov/blast/executables/blast+/

12 http://metavir-meb.univ-bpclermont.fr/index.php?page=Taxo
