# Supplementary material for: MG-Digger: An Automated Pipeline to Search for Giant Virus-Related Sequences in Metagenomes
Source: Front Microbiol. 2016 Mar 31;7:428. doi: 10.3389/fmicb.2016.00428 (PMC4814491; doi:10.3389/fmicb.2016.00428)
Supplement: Supplementary file 1 [file Data_Sheet_1.DOCX]

**Supplementary material**

**SUPPLEMENTARY FIGURES**

**Supplementary Figure S1**. **Phylogenetic tree based on Topoisomerase IIA.**

Sequences used were the metagenome read matching Topoisomerase IIA from *Pithovirus sibericum* and its 20 best BLASTp hits (which included *Pithovirus sibericum* sequence as top hit) in the NCBI GenBank protein sequence database (http://www.ncbi.nlm.nih.gov/protein). These sequences were aligned using the Muscle program (Edgar2004). Phylogenetic reconstructions were performed using maximum likelihood (ML) inference through the Fastree program (Price et al., 2010). Phylogenetic tree was constructed using the MEGA6 program (Tamura et al., 2013).

**Supplementary Figure S2**. **Alignment of a metagenome read and Topoisomerase IIA sequence from *Pithovirus sibericum.***

Alignment was performed using the Muscle program (Edgar2004). The representation was built using the GeneDoc software (http://www.psc.edu/biomed/genedoc).

**Supplementary Figure S3**. **Comparison of sequences detected by the ‘Giant Virus Finder’ tool and MetaDig in 16 soil metagenomes*.***

Number of reads detected by the ‘Giant Virus Finder’ tool was determined using data provided in a previous study (Kerepesi et al., 2015).

GVF, ‘Giant Virus Finder’.

**SUPPLEMENTARY TABLES**

**Supplementary Table S1**. List of metagenomes collected and analyzed in the present study.

**Supplementary Table S2**. List of *Megavirales* representatives and virophages whose sequences were used in the present study.

**REFERENCE LIST**

Edgar, R. C. (2004). MUSCLE: multiple sequence alignment with high accuracy and high throughput. *Nucleic Acids Res.* 32, 1792-1797.

Kerepesi, C., Grolmusz, V. (2015) The "Giant Virus Finder" discovers an abundance of giant viruses in the Antarctic dry valleys. arXiv 1503.05575.

Price, M. N., Dehal, P. S., and Arkin, A. P. (2010). FastTree 2--approximately maximum-likelihood trees for large alignments. *PLoS One.* 5, e9490.

Tamura, K., Stecher, G., Peterson, D., Filipski, A., and Kumar, S. (2013). MEGA6: Molecular Evolutionary Genetics Analysis version 6.0. *Mol. Biol. Evol.* 30, 2725-2729.
