# Supplementary figures and images for: MG-Digger: An Automated Pipeline to Search for Giant Virus-Related Sequences in Metagenomes
Source: Front Microbiol. 2016 Mar 31;7:428. doi: 10.3389/fmicb.2016.00428 (PMC4814491; doi:10.3389/fmicb.2016.00428)

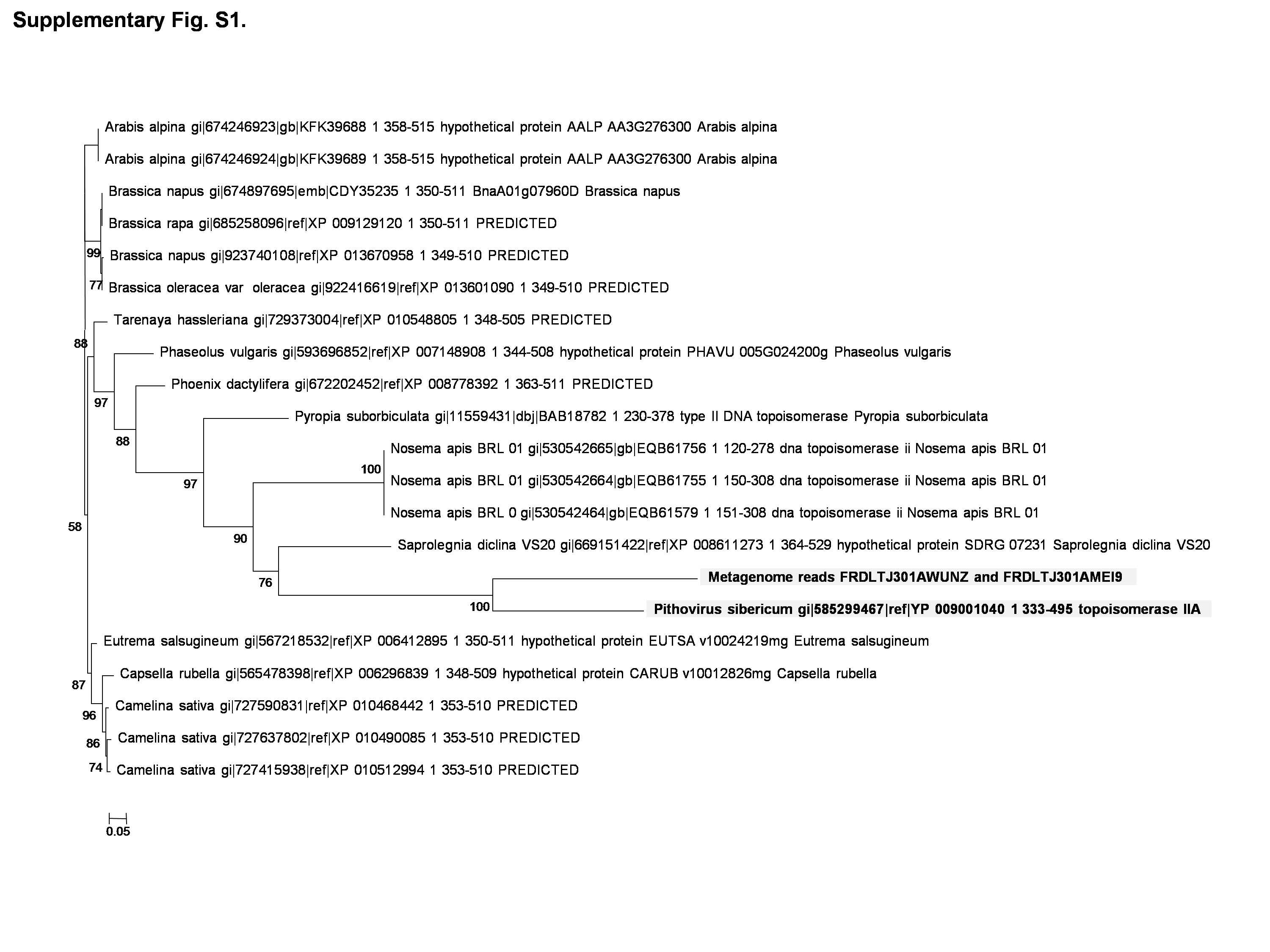

Supplement: Supplementary file 4 [file Image_1.TIF]

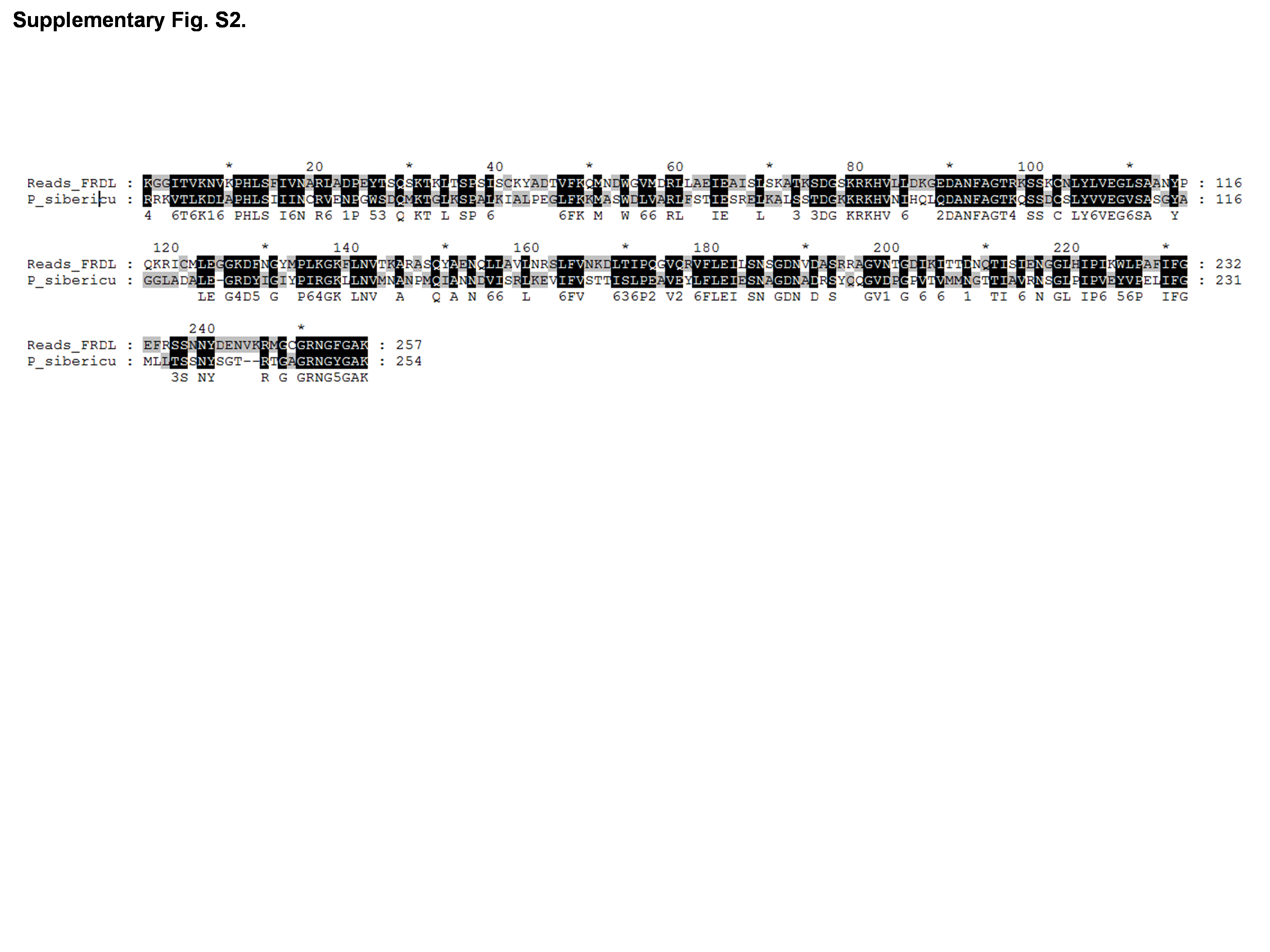

Supplement: Supplementary file 5 [file Image_2.TIF]

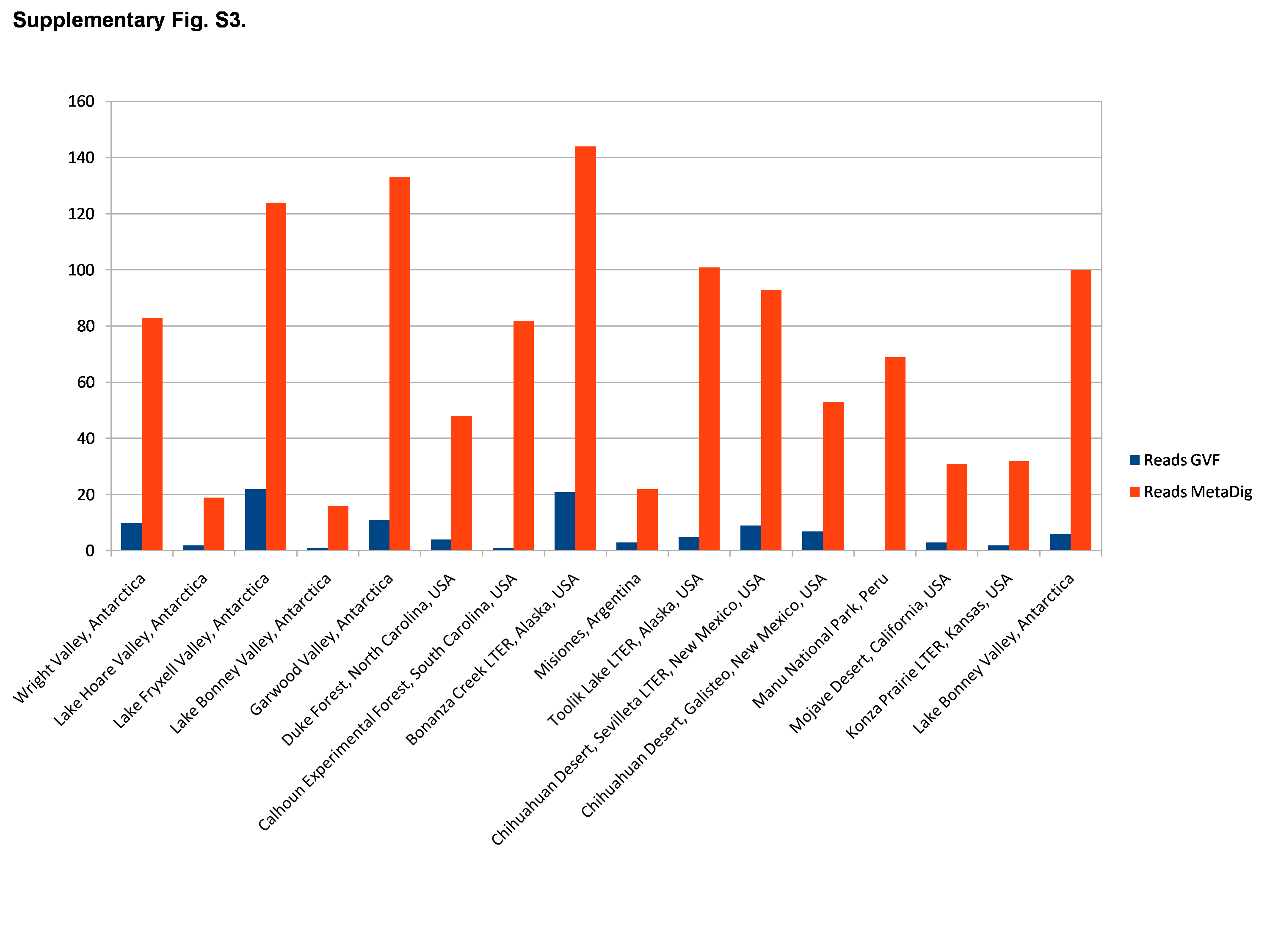

Supplement: Supplementary file 6 [file Image_3.TIF]
